# Integrated Neuro-Evolution-Based Computing Paradigm to Study the COVID-19 Transposition and Severity in Romania and Pakistan

**DOI:** 10.1007/s44196-022-00133-1

**Published:** 2022-09-17

**Authors:** Muhammad Shoaib, Marwan Abukhaled, Saba Kainat, Kottakkaran Sooppy Nisar, Muhammad Asif Zahoor Raja, Ghania Zubair

**Affiliations:** 1 Department of Mathematics, COMSATS University Islamabad, Attock Campus, Attock, Pakistan; 2 Department of Mathematics and Statistics, American University of Sharjah, Sharjah, UAE; 3 Department of Mathematics, College of Arts and Sciences, Prince Sattam bin Abdulaziz University, Wadi Aldawaser, Saudi Arabia; 4 Future Technology Research Center, National Yunlin University of Science and Technology, 123 University Road, Section 3, Douliou, Yunlin 64002 Taiwan, ROC

**Keywords:** Numerical treatment, COVID-19 transmission, Artificial neural networks, Genetic algorithms, Sequential quadratic programming

## Abstract

Numerical treatment of the COVID-19 transposition and severity in Romania and Pakistan has been presented in this study, i.e., ANN-GA-SQP through artificial neural network genetic algorithms (ANN-GA) and sequential quadratic programming (SQP), a design of an integrated computational intelligent paradigm, COVID-19 is widely considered to be the greatest health threat humanity has ever faced. In terms of both health and economics, COVID-19 is a huge disaster. Many academics have looked at the COVID-19 model in their research papers, although they use different traditional techniques to represent it. The use of hybrid suggested solutions to solve this issue in the present article is significant, demonstrating the study's novelty. The SIR model of COVID-19 consists of a susceptible, infectious, and recovered class of population. The activation function for the construction of functions based on fitness in mean squared error sense is developed using nonlinear equations of the COVID-19 SIR model for the best performance of ANN-GA-SQP with the combined potential of GA and SQP of a network. While detailed refining is done with efficient local search with SQP, GAs operates as a global search. In addition, a neuron analysis will be presented to verify the effectiveness and complexity of the proposed method. Adam’s numerical methodology is applied to compare the sustainability and efficacy of the presented paradigm. Analytical evaluations of mean, median, and semi-interquartile range values, as well as Theil’s inequality coefficients, root mean squared error, and mean of absolute deviation) values have been observed. The convergence and correctness of the ANN-GA-SQP approach are further validated by statistical analyses.

## Introduction

Charles Darwin gave the theory of natural selection to define how genetic characteristics may change over time, leading to further generations. Genetic algorithms (GAs) are caused by Darwin's theory [1, 2]. GAs include five steps in their definition of an initial population, fitness function, selection, crossover, and mutation.

The algorithm starts with a population in which an individual’s potential is defined with the help of chromosomes that are made up of genes. The fitness function shows the fitness score of an individual for the strong chance of reproduction. The selection procedure selects the best individual for the next generation. A crossover point is chosen from the parent's DNA to be mated. genes are exchanged among themselves to make offspring until the crossover point is achieved. Mutation happens to maintain a variety of populations and avoid premature events. If the population coincides, then the algorithm will stop working. The GA generates solutions to our problems.

The GA has a wide range of applications in a multitude of sectors, like in task scheduling problems in cloud computing [3], hybrid gene selection approach for cancer classification [4], heat exchanger networks [5], structural crack detection [6], nervous stomach nonlinear model [7], and mosquito dispersal model [8]. The significant potential of the heuristic computing scheme based on stochastic solvers is exploited to solve linear and nonlinear models by using the high predictive potential of artificial neural networks (ANNs) beneath the optimization of global and local search techniques [9–14].

The goal of this research is to examine a SIR model of COVID-19 transposition and severity in Romania and Pakistan and obtain simulation results in order to fully comprehend system behaviour using stochastic methodologies. The stochastic computing model's significant potential is to examine the idea of attempting to solve models by combining the artificial neural network's high generalization potential with the combined abilities of local and global search strategies. Multi-objective problems [15], hybrid cable networks [16], and building model calibration [17] are just a few examples of recent research that has extensively used soft computing approaches.

Recently, scientists used GAs to solve constrained optimization problems [18], optimise the performance of an industrial system [19], pyrolysis kinetics of biomass [20], hybrid ITLHHO algorithm for numerical and engineering optimization problems [21], and hybrid TLNNABC algorithm for reliability optimization and engineering design problems [22].

Coronaviruses are a type of virus that can infect humans as well as animals. Coronaviruses are zoonotic or vector-borne infections, which means they can spread from animals to humans [23]. Human coronavirus can cause symptoms ranging from the common cold to critical disease [24]. The disease acute respiratory syndrome spreads from animal to human. Transfer from animal to human spread by digestive track and breathing droplets while human to human transfer is because of direct contact from corona infected person [25, 26].

The fourth wave of COVID-19 in Romania is highlighted due to the high death rate, which was 500 daily deaths recorded by the middle of October 2021 [27]. In Europe, Romania is first due to high percentiles in cardiovascular disease, hypertension, chronic conditions, and smoking [28]. Romania has a high rate of deaths due to heart disease and cancer [29]. 11.6% of the population has diabetes, and the number doubles for prediabetes, as confirmed by Predator analysis [30] and Mentor analysis [31]. As a result, it is shown that a large percentage of severe acute respiratory syndrome infections (SARS-CoV-2) in Romania will have a severe form of the disease or will be tragic.

COVID-19 causes pneumonia, kidney damage, respiratory distress syndrome, and mortality in severe cases [32]. On February 26, 2020, the first COVID-19 case was confirmed in Pakistan. Following that, Pakistan saw a surge in COVID-19 cases [33]. The WHO has warned that unless sufficient precautions are taken, Pakistan might become the next COVID-19 centre. However, the public's assessment of the risk of COVID-19 infection is unsatisfactory, owing to a lack of compliance with preventive measures. Due to a lack of medical treatment, governments attempted to prevent the spread of the virus by implementing lockdowns and quarantines [34]. the spread of COVID-19, people are advised to stay at home and maintain social distance [35]. In Pakistan, the average number of new infections recorded every day has increased by more than 2,400 in the last 3 weeks, accounting for 43% of the previous record.

In this article, artificial neural networks on the basis of log-sigmoid activation function is incorporated and its optimization for the computation of the nonlinear COVID-19 model in an innovative neuro-evolutionary simulation using the global optimization effectiveness of GAs incorporated with swift global optimization of sequential quadratic programming (SQP), demonstrating the uniqueness of this study. It's a unique piece, and its uniqueness and inventive contributions are described further down.

The uniqueness of this study is explained in two steps:

1. The COVID-19 model is designed using a suitable derivation process.
2. A computing process dependent on machine learning or computational intelligence knacks is employed to address the COVID-19 model using log sigmoid based artificial neural network applications.

The differential equations of COVID-19 SIR model are [36]:

1 $$ \begin{gathered} \frac{{{\text{d}}S}}{{{\text{d}}t}} = \mu - S\mu - \frac{SI\beta }{{vI + 1}} \hfill \\ \frac{{{\text{d}}I}}{{{\text{d}}t}} = \frac{SI\beta }{{vI + 1}} - I(\alpha + \delta + \mu ) \hfill \\ \frac{{{\text{d}}R}}{{{\text{d}}t}} = I\alpha - R\mu . \hfill \\ \end{gathered} $$

The theoretical analysis based validity of the COVID-19 SIR model as portray with three classes based ODE system (1) along with justification of parameters in terms of global and local stability indices are reported in [36]. The system parameters, as well as related variables and explanations, are listed in Tables 1 and 2. While the purpose of this research is to resolve Eq. (1) using ANN-GA-SQP, an intelligent computing paradigm based on GAs and sequential quadratic programming (SQP).

**Table 1:** Demonstration of state variables of SIR model

Parameter	Description
S	susceptible Individuals
I	Infectious Individuals
R	Recovered Individuals

**Table 2:** Detail of involved parameters of SIR model

Parameter	Interpretation	Values
$\mu$	birth and death rate	$0.000041$
$\beta$	transmission rate	$0.097$
$v$	inhibition effect or precautions	$9510$
$\alpha$	recovery rate of infectious individuals	$0.0519$
$\delta$	disease-related death rate	$0.00703$

The primary contributions and insights of the design ANN-GA-SQP scheme are briefly highlighted as follows:

- In such a unique implementation of the dynamical intelligent modelling solver, the stability of ANNs trained at first for global search is dependent on GAs combined with SQP for quick changing of the variables for the solution of the governing equations defining the COVID-19 SIR model.
- The Adam numerical approach and absolute error analysis are used to compare the reliability, durability, and efficacy of the COVID-19 SIR model.
- Statistical assessments of mean absolute deviation (MAD), root mean square error (RMSE), and Theil's inequality coefficient (TIC) weightings from multiple ANN-GA-SQP implementations verify the efficacy verification.

## Solution Methodology

The methodology for solving the COVID-19 SIR model in two steps is as follows:

1. Introducing fitness functions based on error using the ANN-GA-SQP framework.
2. The necessary information is supplied for employing a combination of GA and SQP to boost the fitness function for the proposed model.

**Fig. 1:**
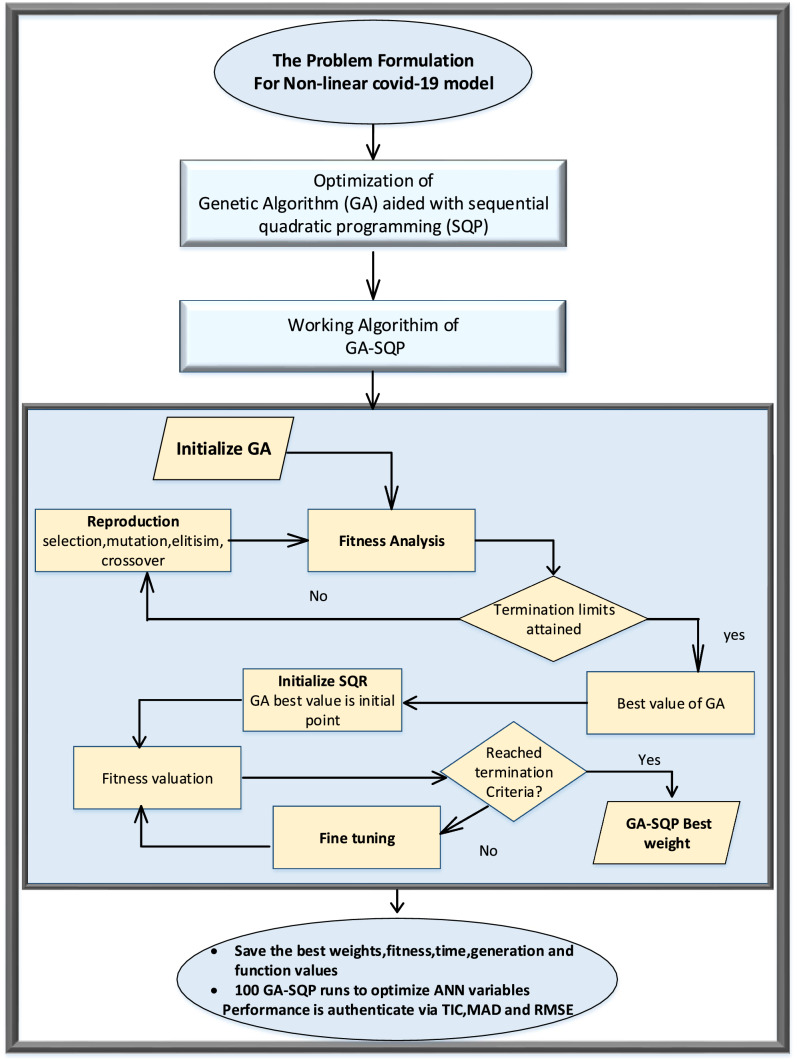
Designed methodology of COVID-19 SIR Model

The ANN-GA-SQP design methodology is presented in Fig. 1.

## Modelling of ANN

The quantitative equations are stated in the perspective of system (1) utilising continual mapping of ANN in the manner of the indicated findings and the nth order derivatives as follows:

2 $$ \begin{gathered} \left[ \begin{gathered} \hat{S}\left( t \right) \hfill \\ \hat{I}\left( t \right) \hfill \\ \hat{R}\left( t \right) \hfill \\ \end{gathered} \right]\,\, = \,\,\,\left[ \begin{gathered} \sum\limits_{i = 1}^{m} {\phi_{S,i} q\left( {\eta_{S,i} t + b_{S,i} } \right)} \hfill \\ \sum\limits_{i = 1}^{m} {\phi_{I,i} q\left( {\eta_{I,i} t + b_{I,i} } \right)} \hfill \\ \sum\limits_{i = 1}^{m} {\phi_{R,i} q\left( {\eta_{R,i} t + b_{R,i} } \right)} \hfill \\ \end{gathered} \right], \hfill \\ \left[ \begin{gathered} \hat{S}^{(n)} \left( t \right) \hfill \\ \hat{I}^{(n)} \left( t \right) \hfill \\ \hat{R}^{(n)} \left( t \right) \hfill \\ \end{gathered} \right]\,\, = \,\,\,\left[ \begin{gathered} \sum\limits_{i = 1}^{m} {\phi_{S,i} q^{(n)} \left( {\eta_{S,i} t + b_{S,i} } \right)} \hfill \\ \sum\limits_{i = 1}^{m} {\phi_{I,i} q^{(n)} \left( {\eta_{I,i} t + b_{I,i} } \right)} \hfill \\ \sum\limits_{i = 1}^{m} {\phi_{R,i} q^{(n)} \left( {\eta_{R,i} t + b_{R,i} } \right)} \hfill \\ \end{gathered} \right], \hfill \\ \end{gathered} $$

where the weight vector is denoted by "W" having an unknown value, given as

$$ \begin{gathered} W = \left[ {W_{S} ,W_{I} ,\,W_{R} } \right], \hfill \\ W_{S} = \left[ {\phi_{S} ,\eta_{S} ,b_{S} } \right],W_{I} = \left[ {\phi_{I} ,\eta_{I} ,b_{I} } \right],W_{R} = \left[ {\phi_{R} ,\eta_{R} ,b_{R} } \right], \hfill \\ \phi_{S} = \left[ {\phi_{S,1} ,\,\,\,\phi_{S,2} ,\,\,\,\phi_{S,3} , \ldots ,\phi_{S,m} } \right],\phi_{I} = \left[ {\phi_{I,1} ,\phi_{I,2} ,\phi_{I,3} , \ldots ,\phi_{I,m} } \right], \hfill \\ \phi_{R} = \left[ {\phi_{R,1} ,\phi_{R,2} ,\phi_{R,3} , \ldots ,\phi_{R,m} } \right], \hfill \\ \eta_{S} = \left[ {\eta_{S,1} ,\eta_{S,2} ,\eta_{S,3} , \ldots ,\eta_{S,m} } \right],\eta_{I} = \left[ {\eta_{I,1} ,\eta_{I,2} ,\eta_{I,3} , \ldots ,\eta_{I,m} } \right], \hfill \\ \eta_{R} = \left[ {\eta_{R,1} ,\eta_{R,2} ,\eta_{R,3} , \ldots ,\eta_{R,m} } \right], \hfill \\ b_{S} = \left[ {b_{S,1} ,b_{S,2} ,b_{S,3} , \ldots ,b_{S,m} } \right],b_{I} = \left[ {b_{I,1} ,b_{I,2} ,b_{I,3} , \ldots ,b_{I,m} } \right], \hfill \\ b_{R} = \left[ {b_{R,1} ,b_{R,2} ,b_{R,3} , \ldots ,b_{R,m} } \right], \hfill \\ \end{gathered} $$

Applying the log-sigmoid function given as $q(t) = (1 + e^{ - t} )^{ - 1}$. The system given in (2) can correspond as follows:

3 $$ \begin{gathered} \left[ \begin{gathered} \hat{S}\left( t \right) \hfill \\ \hat{I}\left( t \right) \hfill \\ \hat{R}\left( t \right) \hfill \\ \end{gathered} \right]\,\, = \,\,\,\left[ \begin{gathered} \sum\limits_{i = 1}^{m} {\frac{{\phi_{S,i} }}{{1 + e^{{ - \left( {\eta_{S,i} t + b_{S,i} } \right)}} }}} \, \hfill \\ \sum\limits_{i = 1}^{m} {\frac{{\phi_{I,i} }}{{1 + e^{{ - \left( {\eta_{I,i} t + b_{I,i} } \right)}} }}} \hfill \\ \sum\limits_{i = 1}^{m} {\frac{{\phi_{R,i} }}{{1 + e^{{ - \left( {\eta_{R,i} t + b_{R,i} } \right)}} }}} \hfill \\ \end{gathered} \right], \hfill \\ \left[ \begin{gathered} \hat{S}^{\prime}\left( t \right) \hfill \\ \hat{I}^{\prime}\left( t \right) \hfill \\ \hat{R}^{\prime}\left( t \right) \hfill \\ \end{gathered} \right]\,\, = \,\,\,\left[ \begin{gathered} \sum\limits_{i = 1}^{m} {\frac{{\eta_{S,i} \phi_{S,i} e^{{ - \left( {\eta_{S,i} t + b_{S,i} } \right)}} }}{{\left( {1 + e^{{ - \left( {\eta_{S,i} t + b_{S,i} } \right)}} } \right)}}} \,\, \hfill \\ \sum\limits_{i = 1}^{m} {\frac{{\eta_{I,i} \phi_{I,i} e^{{ - \left( {\eta_{I,i} t + b_{I,i} } \right)}} }}{{\left( {1 + e^{{ - \left( {\eta_{I,i} t + b_{I,i} } \right)}} } \right)}}} \hfill \\ \sum\limits_{i = 1}^{m} {\frac{{\eta_{R,i} \phi_{R,i} e^{{ - \left( {\eta_{R,i} t + b_{R,i} } \right)}} }}{{\left( {1 + e^{{ - \left( {\eta_{R,i} t + b_{R,i} } \right)}} } \right)}}} \hfill \\ \end{gathered} \right]. \hfill \\ \end{gathered} $$

Using the network design (3) described above, we get function e based on error in the perspective of mean squared error (MSE):

4 $$ e = e_{1} + e_{2} + e_{3} + e_{4} , $$

5 $$ e_{1} = \frac{1}{N}\sum\limits_{m = 1}^{N} {\left( {\hat{S}^{\prime}_{m} - \mu + \frac{{\beta \hat{I}_{m} \hat{S}_{m} }}{{1 + \nu \hat{I}_{m} }} + \mu \hat{S}_{m} } \right)} , $$

6 $$ e_{2} = \frac{1}{N}\sum\limits_{m = 1}^{N} {\left( {\hat{I}^{\prime}_{m} - \frac{{\beta \hat{I}_{m} \hat{S}_{m} }}{{1 + \nu \hat{I}_{m} }} + (\alpha + \mu + \delta )\hat{I}_{m} } \right)} , $$

7 $$ e_{3} = \frac{1}{N}\sum\limits_{m = 1}^{N} {\left( {\hat{R}^{\prime}_{m} - \alpha_{2} \hat{I}_{m} + \mu \hat{R}_{m} } \right)} , $$

8 $$ e_{4} = \frac{1}{3}\left( {\left( {\hat{S}_{0} - I_{1} } \right)^{2} + \left( {\hat{I}_{0} - I_{2} } \right)^{2} + \left( {\hat{R}_{0} - I_{3} } \right)^{2} } \right), $$

where $Nh = 1,\,\,t_{m} = mh,\,\hat{S}_{m} = \hat{S}(t_{m} ),\,\hat{I}_{m} = \hat{I}(t_{m} )\,{\text{and}}\,\hat{R}_{m} = \hat{R}(t_{m} )$. The susceptible individuals, infectious individuals, and recovered individuals, respectively, are denoted as $\hat{S}_{m} ,\,\hat{I}_{m} \,{\text{and }}\hat{R}_{m}$ respectively. The SIR model's initial conditions-based fitness function is represented by differential equations error functions. The best available weights can be used to estimate the findings, as long as the error function shown in Eq. 4 approaches zero. Hence the suggested solutions $\left[ {\hat{S}\left( t \right),\,\,\hat{I}\left( t \right),\,\,\,\hat{R}\left( t \right)\,} \right]$ are similar to the exact results, i.e., $\left[ {\hat{S}\left( t \right) \to S\left( t \right)} \right]$, $\left[ {\,\hat{I}\left( t \right) \to I\left( t \right)} \right]$, and $\left[ {\,\hat{R}\left( t \right) \to R\left( t \right)} \right]$.

## Optimization Process of ANN-GA-SQP Approach

To improve the error-based function (4) expressing the COVID-19 SIR model, the relevant information for GA-SQP hybridization is given.

An artificial neural network (ANN) is a biologically inspired computational model made up of hundreds of artificial neurons that are linked together by weights to form the neural structure and process data. Artificial neural networks include six properties that make them more trustworthy than other techniques: network architecture, collective solution, parallel processing, fault tolerance, distributed memory, and learning ability.

Genetic algorithms are made up of natural genetic and natural selection mechanisms as well as certain important concepts from genetics, in order to artificially build an optimization approach. The GA used in this research is available in the MATLAB optimization toolbox. The GA is a practical and dependable global search optimization strategy that employs initial population, fitness function, selection, crossover, and mutation. The fittest people are picked, and a new population is formed in order to increase the quality of the solution.

Recently, the GA has been used in a number of well-known optimization procedures, including optimal parameters for stereo lithography processes [37], heart disease diagnosis [38], power transformer fault diagnosis [39], breast cancer diagnosis [40], cluster analysis [41], and utility bidding strategies for the competitive marketplace [42].

Sequential quadratic programming (SQP) is a popular and dependable nonlinear continuous optimization method. It starts with a single point and finds a solution using gradient information. SQP demands a big initial solution to maximise the possibilities of getting an acceptable solution and avoid the local optima. Sequential quadratic programming (SQP), a local search approach used for fine-tuning GA approximations, improves the GA process even further. SQP has been used to solve a variety of problems, including singular Thomas–Fermi systems [43], fourth order nonlinear Emden–Fowler equations [44], MIMO Feedback Control System Design [45], and dengue fever SIR model [46].

This study proposes ANNs based networks to model the COVID-19 SIR differential system as portrayed in solution methodology section. The training/learning of the weights of the networks are carried out with integrated optimization mechanism, i.e., the GA-SQP based hybrid algorithm, which combines optimization knacks of both GA and SQP. The GA algorithm is a global algorithm that performs well in a large search space and in a good exploration scheme. The SQP method, on the other hand, is effective at finding local optima for restricted nonlinear optimization problems by taking the best weights of GA as a start-point, but in standalone operating mode it cannot ensure that the solution search achieves the global optimum. Therefore, GA-SQP starts by determining the global optimum (GA operation) throughout the whole solution region and then quickly adjust the parameter via local search (SQP operation). The integration of GA-SQP considerably improves optimization strength than that of standalone optimization of either GAs and SQP algorithm and accordingly achieved the better solution quality, stability and convergence to the optimal solution. The proposed hybrid approach eliminates the need for a suitable starting point and provides a faster convergence speed and increased convergence accuracy in order to find the best solution.

For the solution of the COVID-19 SIR model, the technique is carried out in MATLAB software utilising GA-SQP hybridization to identify the unknown weight of ANNs (1). The pseudocode of the ANN-GA-SQP optimization procedure representing the COVID-19 SIR model is presented in Table 3.

**Table 3:**
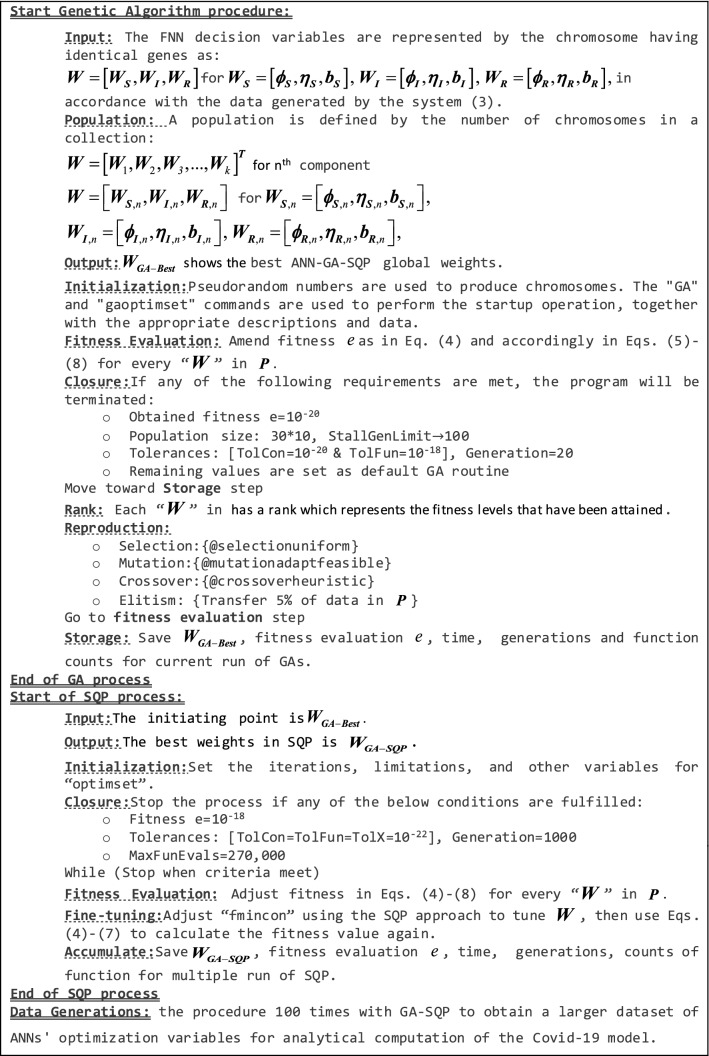
Pseudocode of ANN-GA-SQP optimization method for COVID-19 SIR model

## Performance Grades

In computing the nonlinear COVID-19 SIR model described in Eq. 1, the Theil's inequality coefficient (TIC), root mean square error (RMSE), and Mean of absolute deviation (MAD) are efficiency estimates. Algebraic formulations for such operators is as follows:

9 $$ \left[ \begin{gathered} {\text{RMSE}}_{S} \hfill \\ {\text{RMSE}}_{I} \hfill \\ {\text{RMSE}}_{R} \hfill \\ \end{gathered} \right]\, = \,\left[ \begin{gathered} \sqrt {\frac{1}{m}\sum\limits_{i = 1}^{m} {\left( {S_{i} - \hat{S}_{i} } \right)}^{2} } \hfill \\ \sqrt {\frac{1}{m}\sum\limits_{i = 1}^{m} {\left( {I_{i} - \hat{I}_{i} } \right)}^{2} } \hfill \\ \sqrt {\frac{1}{m}\sum\limits_{i = 1}^{m} {\left( {R_{i} - \hat{R}_{i} } \right)}^{2} } \hfill \\ \end{gathered} \right], $$

10 $$ \left[ \begin{gathered} {\text{TIC}}_{S} \hfill \\ {\text{TIC}}_{I} \hfill \\ {\text{TIC}}_{R} \hfill \\ \end{gathered} \right]\, = \,\left[ \begin{gathered} \frac{{\sqrt {\frac{1}{m}\sum\limits_{i = 1}^{m} {\left( {\left( {S_{i} - \hat{S}_{i} } \right)} \right)}^{2} } }}{{\left( {\sqrt {\frac{1}{m}\sum\limits_{i = 1}^{m} {S_{i}^{2} } } + \sqrt {\frac{1}{m}\sum\limits_{i = 1}^{m} {\hat{S}_{i}^{2} } } } \right)}} \hfill \\ \frac{{\sqrt {\frac{1}{m}\sum\limits_{i = 1}^{m} {\left( {I_{i} - \hat{I}_{i} } \right)}^{2} } \,}}{{\left( {\sqrt {\frac{1}{m}\sum\limits_{i = 1}^{m} {I_{i}^{2} } } \, + \sqrt {\frac{1}{m}\sum\limits_{i = 1}^{m} {\hat{I}_{i}^{2} } } \,} \right)}} \hfill \\ \frac{{\sqrt {\frac{1}{m}\sum\limits_{i = 1}^{m} {\left( {R_{i} - \hat{R}_{i} } \right)}^{2} } }}{{\left( {\sqrt {\frac{1}{m}\sum\limits_{i = 1}^{m} {R_{i}^{2} } } + \sqrt {\frac{1}{m}\sum\limits_{i = 1}^{m} {\hat{R}_{i}^{2} } } } \right)}} \hfill \\ \end{gathered} \right], $$

11 $$ \left[ \begin{gathered} {\text{MAD}}_{S} \hfill \\ {\text{MAD}}_{I} \hfill \\ {\text{MAD}}_{R} \hfill \\ \end{gathered} \right]\, = \,\left[ \begin{gathered} \frac{1}{m}\sum\limits_{i = 1}^{m} {\left| {S_{i} - \hat{S}_{i} } \right|} \hfill \\ \frac{1}{m}\sum\limits_{i = 1}^{m} {\left| {I_{i} - \hat{I}_{i} } \right|} \hfill \\ \frac{1}{m}\sum\limits_{i = 1}^{m} {\left| {R_{i} - \hat{R}_{i} } \right|} \hfill \\ \end{gathered} \right]. $$

## Results and Discussion

This section offers detailed explanations of the COVID-19 SIR model's acquired findings in the system of Eq. (1).The Adams method's findings support the validity of the ANN-GA-SQP modelling technique. Analytical results are also supplied to guarantee that the proposed approach is exact and correct. Using the data from Table 2, the new form of the COVID-19 SIR model is represented in Eq. (1) as:

12 $$ \left\{ \begin{gathered} \frac{{{\text{d}}S}}{{{\text{d}}t}} = 0.000041 - \frac{0.0971IS}{{1 + 9510I}} - 0.000041S,\quad \quad \quad \quad \quad \quad \quad \quad S\left( 0 \right) = 0.999933827, \hfill \\ \frac{{{\text{d}}I}}{{{\text{d}}t}} = \frac{0.0971IS}{{1 + 9510I}} - (0.0519 + 0.000041 + 0.00703)I,\quad \quad \quad \quad I\left( 0 \right) = 0.0000506045, \hfill \\ \frac{{{\text{d}}R}}{{{\text{d}}t}} = 0.0519I - 0.000041R,\quad \quad \quad \quad \quad \quad \quad \quad \quad \quad \quad \quad \quad R\left( 0 \right) = 0.0000155682. \hfill \\ \end{gathered} \right. $$

For the system of Eq. (12), the fitness function is explained as:

13 $$ \begin{aligned} e & = \frac{1}{N}\sum\limits_{m = 1}^{N} {\left( \begin{gathered} \left[ {\hat{S}^{\prime}_{m} - 0.000041 + \frac{{0.0971\hat{I}_{m} \hat{S}_{m} }}{{1 + 9510\hat{I}_{m} }} + 0.000041\hat{S}_{m} } \right]^{2} + \hfill \\ + \left[ {\hat{I}^{\prime}_{m} - \frac{{0.0971\hat{I}_{m} \hat{S}_{m} }}{{1 + 9510\hat{I}_{m} }} + (0.0519 + 0.000041 + 0.00703)\hat{I}_{m} } \right]^{2} \hfill \\ + \left[ {\hat{R}^{\prime}_{m} - 0.0519\hat{I}_{m} + 0.000041\hat{R}_{m} } \right]^{2} \hfill \\ \end{gathered} \right)} \\ & \quad + \frac{1}{3}\left( {\left( {\hat{S}_{0} - 0.999933827} \right)^{2} + \left( {\hat{I}_{0} - 0.0000506045} \right)^{2} + \left( {\hat{R}_{0} - 0.0000155682} \right)^{2} } \right), \\ \end{aligned} $$

14 $$ \begin{gathered} \hat{S}\left( t \right) = \frac{ - 0.0217}{{1 + e^{{ - \left( { - 0.0770t + 0.9549} \right)}} }} - \frac{0.5837}{{1 + e^{{ - \left( {1.5730t - 0.4275} \right)}} }} - \frac{0.788}{{1 + e^{{ - \left( { - 0.3834t + 0.6229} \right)}} }} + \frac{0.1979}{{1 + e^{{ - \left( {0.3224t - 1.1143} \right)}} }} \hfill \\ \,\,\,\,\,\,\,\,\,\,\,\,\, + \frac{0.1575}{{1 + e^{{ - \left( { - 0.5454t + 1.0931} \right)}} }}, \hfill \\ \end{gathered} $$

15 $$ \begin{gathered} \hat{I}\left( t \right) = \frac{ - 0.6820}{{1 + e^{{ - \left( {0.4332t + 1.2957} \right)}} }} - \frac{1.0691}{{1 + e^{{ - \left( {0.5241t + 1.4174} \right)}} }} + \frac{0.2961}{{1 + e^{{ - \left( {0.6874t + 1.9224} \right)}} }} + \frac{0.8264}{{1 + e^{{ - \left( { - 1.3129t + 0.8291} \right)}} }} \hfill \\ \,\,\,\,\,\,\,\,\,\,\,\,\,\,\, + \frac{0.1954}{{1 + e^{{ - \left( {0.5725t + 1.6519} \right)}} }}, \hfill \\ \end{gathered} $$

16 $$ \begin{gathered} \hat{R}\left( t \right) = \frac{2.3490}{{1 + e^{{ - \left( { - 0.3377t - 0.0004} \right)}} }} - \frac{0.3166}{{1 + e^{{ - \left( {1.6186t + 0.9567} \right)}} }} - \frac{0.9999}{{1 + e^{{ - \left( { - 0.2528t + 0.9209} \right)}} }} + \frac{0.8834}{{1 + e^{{ - \left( {0.4910t + 1.8027} \right)}} }} \hfill \\ \,\,\,\,\,\,\,\,\,\,\,\,\,\, - \frac{1.4639}{{1 + e^{{ - \left( { - 1.2933t - 0.0008} \right)}} }}, \hfill \\ \end{gathered} $$

**Fig. 2:**
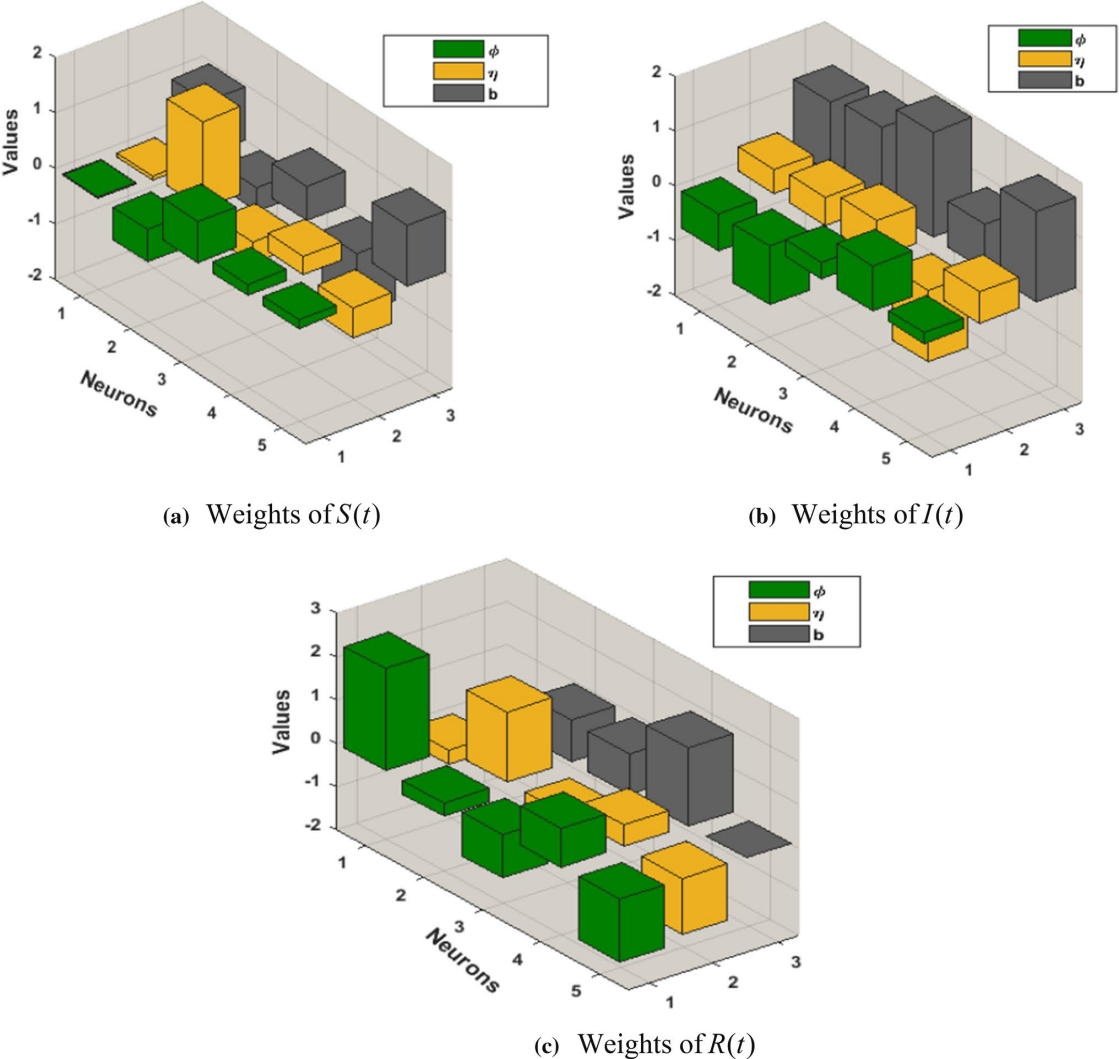
Weights of ANN-GA-SQP based on the fitness for COVID-19 SIR model

For 100 iterations, the GA-SQP combination approach optimises the COVID-19 SIR model explained in Eq. (1) to produce variables of the ANN framework employing 5 neurons for the computation. In Fig. 2, the ANN weights computed by GA-SQP are presented, and these weights are employed in the first equation of (3) to give approximated quantitative data for Eq. (1) for 5 neurons.

**Fig. 3:**
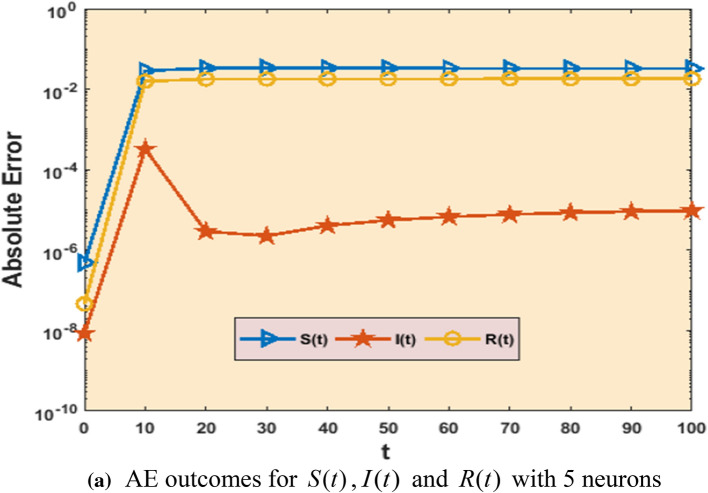
Analysis about absolute error outcomes for five numbers of neurons

**Fig. 4:**
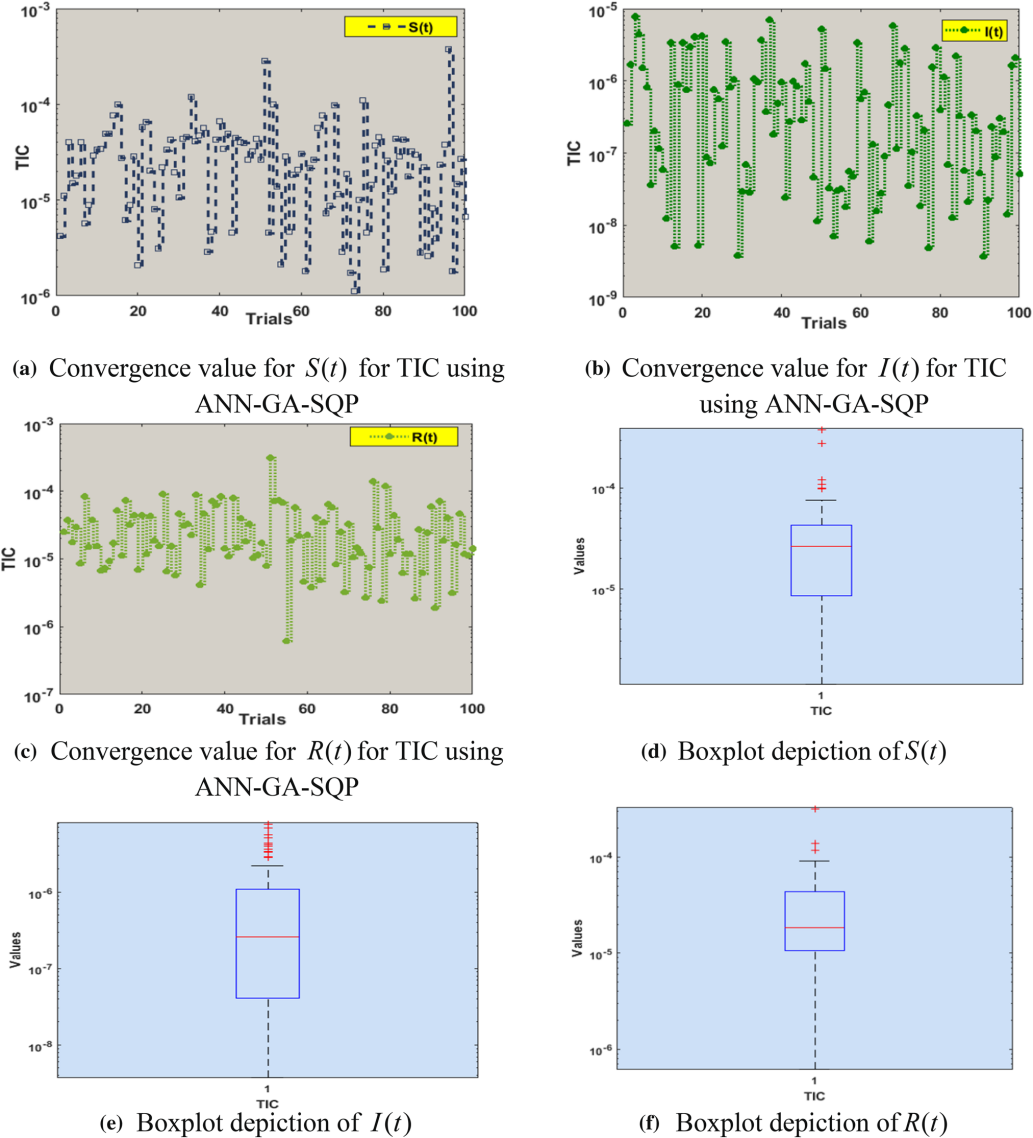
Performance analysis of TIC for *S*(*t*), $I(t)$, $R(t)$ with boxplots including 5 neurons using ANN-GA-SQP for COVID-19 SIR model

**Fig. 5:**
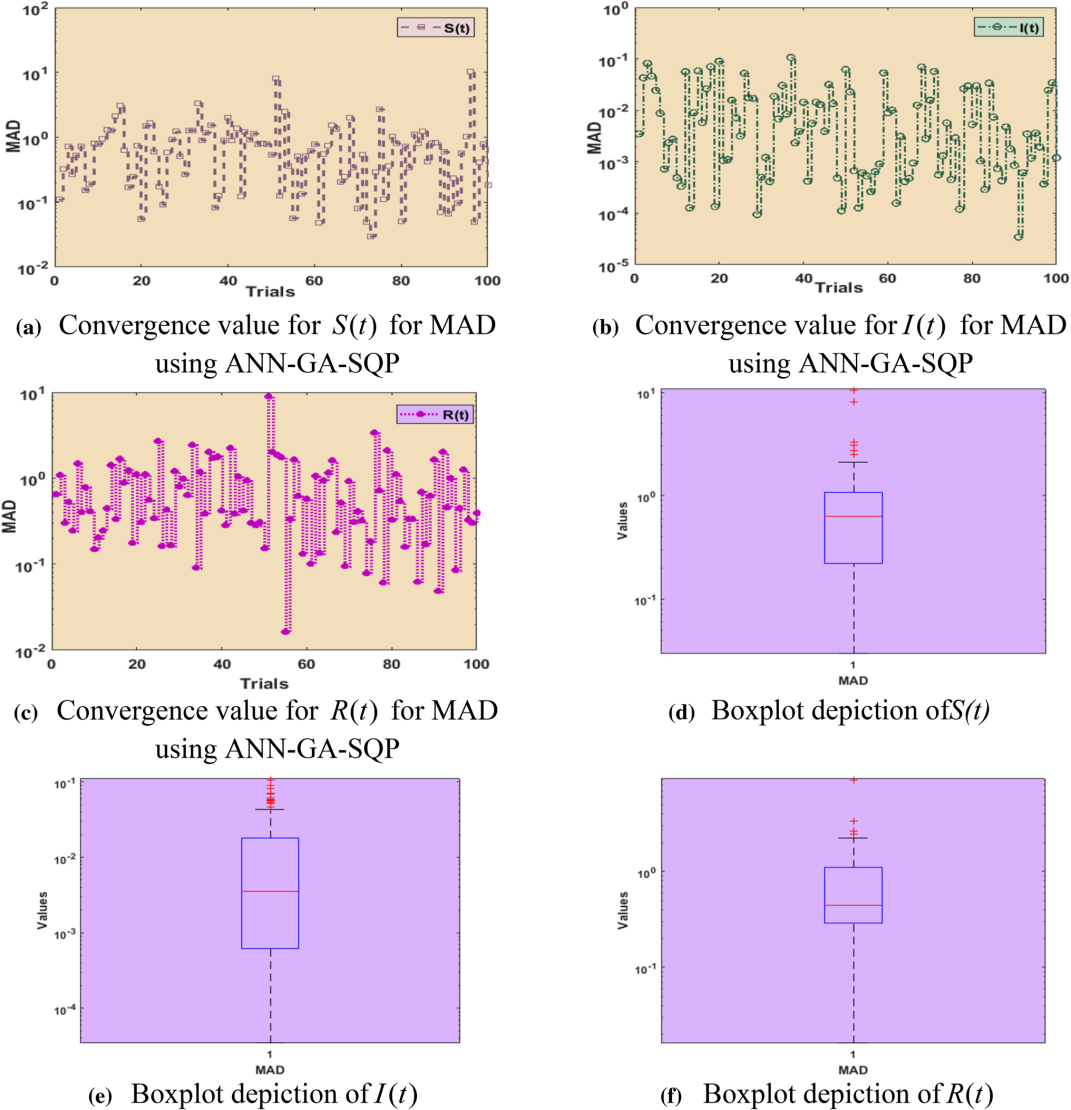
Performance analysis of MAD for *S*(*t*), $I(t)$, $R(t)$ with boxplots including 5 neurons using ANN-GA-SQP for COVID-19 SIR model

**Fig. 6:**
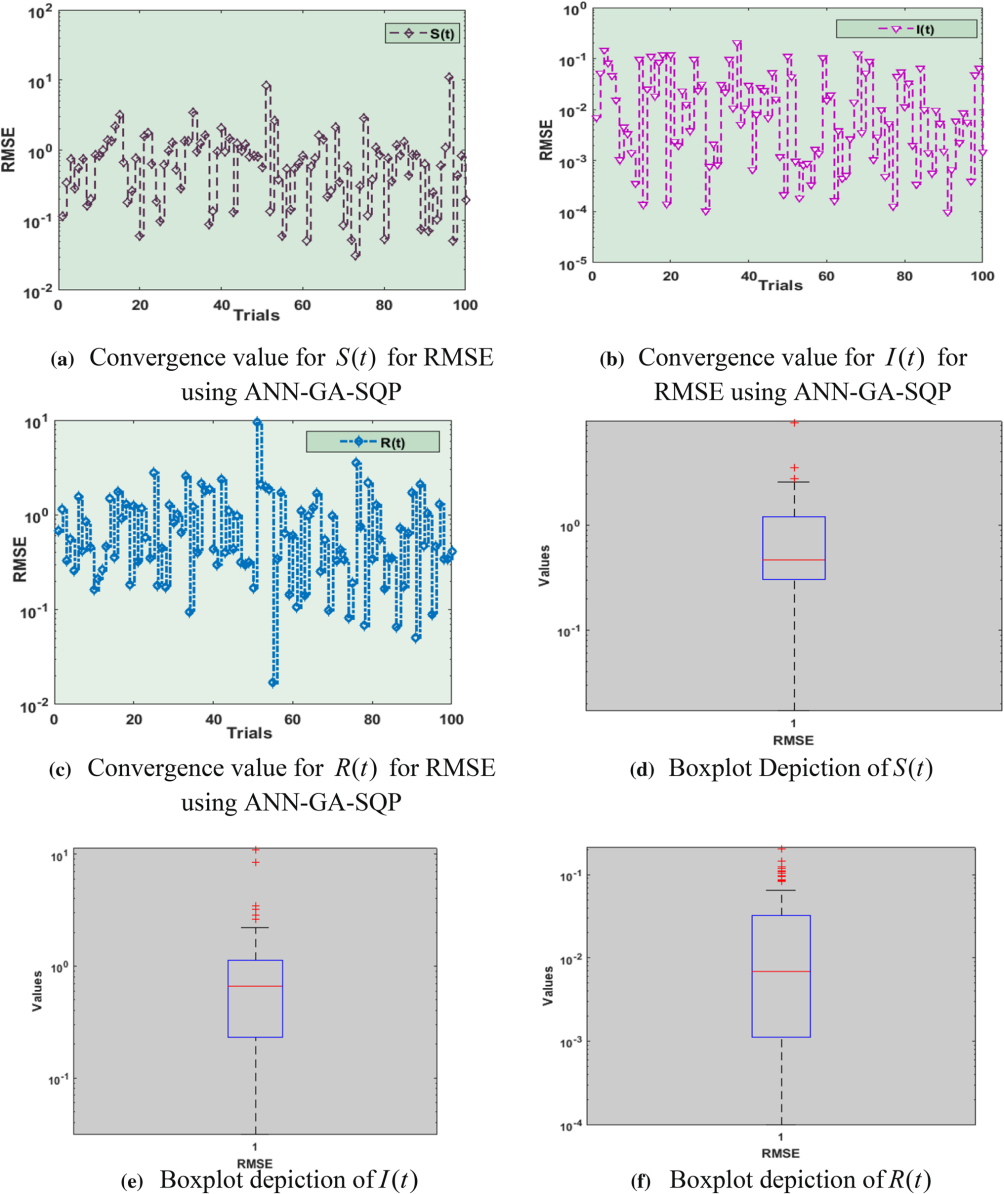
Performance analysis of RMSE for *S*(*t*), $I(t)$, $R(t)$ with boxplots, including 5 neurons using ANN-GA-SQP for COVID-19 SIR model

**Fig. 7:**
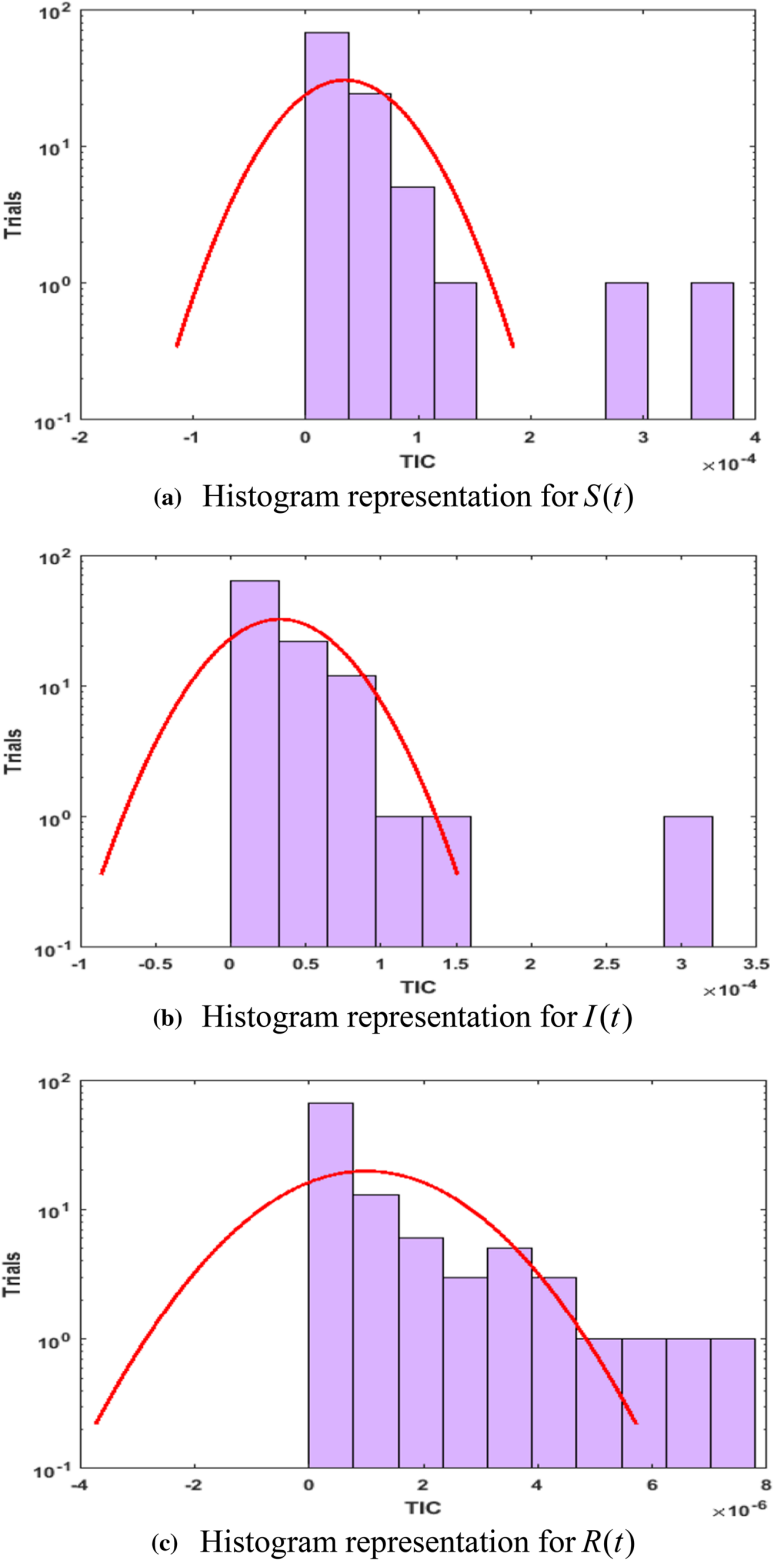
Histograms depiction of TIC for *S*(*t*), $I(t)$, $R(t)$ using ANN-GA-SQP

Equations (14–16) are used with the ANN-GA-SQP to generate the outcome of the COVID-19 model described in Eq. (12).Figs. 2, 3, 4, 5, 6, and 7 with five neurons show the graphical findings for the COVID-19 SIR model. The graph of training weights for a 5 neuron-based ANN framework is shown in Fig. 2a–c for $S(t)$, $I(t)$ and $R(t)$ accordingly. Figure 3 shows the absolute error plots depending on the Adams technique errors for $S(t)$, $I(t)$ and $R(t)$. In Fig. 3 the absolute error values of $S(t)$, $I(t)$ and $R(t)$ lie between 10^–8^ to 10^–6^ and 10^–9^ to 10^–8^ and 10^–8^ to 10^–6^.

The numerical findings for TIC, RMSE, and MAD values, as well as boxplot demonstrations, are shown in Figs. 4, 5, and 6. In Fig. 7, TIC results for an error histogram are depicted. TIC performance values are shown in Fig. 4a–c for the parameters $S(t)$, $I(t)$ and $R(t)$ and their statistical values lie between 10^–6^ to 10^–3^, 10^–9^ to 10^–5^, 10^–7^ to 10^−3^, respectively. In Fig. 4d–f the box plots of all three parameters for TIC are displayed. Values of boxplots for $S(t)$, $I(t)$ and $R(t)$ are between 10^–5^ to 10^–4^, 10^–8^ to 10^–6^, 10^–6^ to 10^−4^, respectively.

Figure 5a–c shows the MAD performance outcomes for the parameters $S(t)$, $I(t)$ and $R(t)$ and their statistical values lie between 10^–2^ to 10^–2^, 10^–5^ to 10^0^, 10^–2^ to 10^1^, respectively. In Fig. 5d–f the box plots of all five parameters for MAD are displayed. Values of boxplots for $S(t)$,$I(t)$ and $R(t)$ are between 10^–1^ to 10^1^, 10^–4^ to 10^–1^, 10^–1^ to 10^0^, respectively.

Figure 6a–c shows the RMSE performance results for the parameters $S(t)$, $I(t)$ and $R(t)$ and their statistical values lie between 10^–2^ to 10^2^, 10^–5^ to 10^0^, 10^–2^ to 10^1^, respectively. In Fig. 6d–f the box plots of all five parameters for RMSE are displayed. of Boxplots for $S(t)$, $I(t)$ and $R(t)$ are between 10^–1^ to 10^0^, 10^–1^ to 10^1^, 10^–4^ to 10^−1^, respectively.

**Fig. 8:**
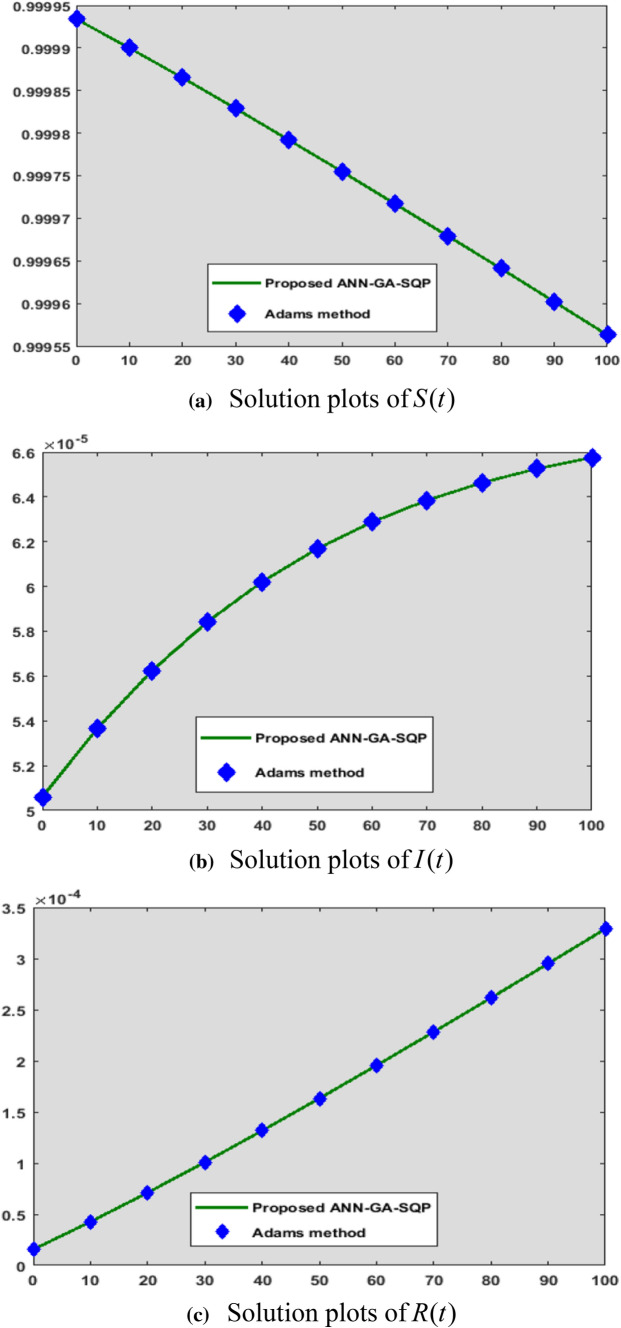
Comparison Analysis of ANN-GA-SQP with Adam numerical technique for COVID-19 SIR model

Figure 7a–c shows the histograms depiction of TIC for *S*(*t*), $I(t)$, $R(t)$ whereas for histogram value for all five parameters are from 10^–5^ to 10^–4^, 10^–5^ to 10^–4^, 10^–7^ to 10^–6^ against trails of 10^–1^ to 10^2^, 10^–1^ to 10^2^, 10^–1^ to 10^2^, respectively. All of these statistical values are approaching zero, indicating the precision and accuracy of the ANN-GA-SQP solution. In Fig. 8, a comparison of ANN-GA-SQP with Adam's numerical technique for the COVID-19 model is shown. The integrated approach for the COVID-19 model is confirmed, validated, and perfected by comparative studies of reference data on stability, accuracy, convergence, and reliability criteria, confirming the study’s innovation and uniqueness. In the future, we will do a comparison of ANN-GA-SQP with deep belief network [47] and convolution neural network [48].

## Concluding Remarks

The quality of an ANN's estimate ability is improved by combining the global search capabilities of GAs with rapid local parameter modifications utilising SQP to resolve a nonlinear COVID-19 SIR model. The COVID-19 SIR model's behaviour is effectively assessed using the ANN-GAS-QP statistical approach, which uses layer-structured neural networks with five neurons. The ANN-GA-SQP numerical results are compared to numerical data collected using the Adams technique, proving the accuracy of the COVID-19 model. With 100 ANN-GA-SQP runs, analytical evaluations of TIC, root mean squared error, and MAD values validate the results. Absolute errors lie around for all the classes of the COVID-19 model. The depiction of discoveries on a continuous array of inputs throughout the training period, smooth application, conceptual simplicity, dependability, application, and modifiability are all advantages of intelligence programming employing ANN-GA-SQP.

In the coming era, the ANN-GA framework is a better alternative to investigate for providing outstanding output from difficult nonlinear issues involving fractional analysis [49], three-point second-order boundary value problems analysis [50], Bouc–Wen hysteresis model [51], nonlinear Emden–Fowler equation [52], and nanofluidic problems [53]. ANN-GA-SQP can also be used in Face Intelligent Perception Technology Integrating Deep Learning under Different Illumination Intensities [54], Vehicle Automatic Driving Target Perception Technology Based on Improved MSRPN Algorithm [55], Research on Robot Path Perception and Optimization Technology based on whale optimization algorithm [56], fractional COVID-19 model [57], risk factors in HIV Transmission: [58], model of immobilized enzyme [59] and deep learning applications[60].The solver's precision and efficiency will be improved using statistical heuristics techniques combined with local search methodology.

## Data Availability

Not applicable.

## Abbreviations

Gas: Genetic algorithms
SQP: Sequential quadratic programming
ANN-GA: Artificial neural network genetic algorithms
TIC: Theil’s inequality coefficient
MAD: Mean absolute deviation
RMSE: Root mean square error
